# Causal relationship between insomnia and thyroid disease: A bidirectional Mendelian randomization study

**DOI:** 10.1002/brb3.70046

**Published:** 2024-09-18

**Authors:** Zhonghui Li, Zonghang Jia, Peng Zhou, Qingqing He

**Affiliations:** ^1^ First Clinical Medical College Shandong University of Traditional Chinese Medicine Jinan China; ^2^ Department of Thyroid and Breast Surgery The 960th Hospital of PLA Joint Logistics Support Force Jinan Shandong China

**Keywords:** causal correlation, insomnia, Mendelian randomization, thyroid disease

## Abstract

**Objective:**

Some correlations between thyroid disorders and insomnia have been found in previous studies; however, the causal relationship between them is unclear. The aim of this study was to investigate the causal relationship between insomnia and five thyroid disorders (hyperthyroidism, hypothyroidism, thyroiditis, thyroid nodules, and thyroid cancer).

**Methods:**

We assessed the causal relationship between insomnia and thyroid disorders using inverse variance weighted, weighted median, and Mendelian randomization (MR)‐Egger analyses in MR analyses and then used inverse MR analyses to assess the causal relationship between thyroid disorders and insomnia.

**Results:**

MR analysis showed that insomnia did not increase the risk of hyperthyroidism, hypothyroidism, thyroiditis, thyroid nodules, and thyroid cancer. However, reverse MR analysis showed that thyroid cancer increased the risk of insomnia (OR = 1.01, 95%CI: 1.00–1.02, *p* = .01), and the other four thyroid disorders had no direct causal relationship with insomnia. Sensitivity analyses indicated that the results were robust and no pleiotropy or heterogeneity was detected.

**Conclusion:**

This study did not find evidence of a bidirectional causal relationship between genetically predicted insomnia and hyperthyroidism, hypothyroidism, thyroiditis, and thyroid nodules. However, we found that although insomnia does not increase the risk of thyroid cancer, thyroid cancer does increase the risk of insomnia.

## Graphical abstract

This MR study found that there is no evidence of a bidirectional causal relationship between gene predicted insomnia and hyperthyroidism, hypothyroidism, thyroiditis, and thyroid nodules, but thyroid cancer may increase the risk of insomnia.

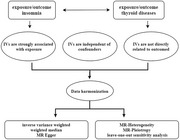

## 1 INTRODUCTION

Thyroid disorders are common endocrine system diseases. Currently, common clinical thyroid disorders include hyperthyroidism, hypothyroidism, thyroiditis, thyroid nodules, and thyroid cancer. Previous epidemiologic studies have shown that approximately 200 million people worldwide suffer from thyroid disease, and the prevalence has continued to increase in recent years (The Lancet, 2012). One such study of Americans found a prevalence of 1% for hyperthyroidism and 5% for hypothyroidism (Vanderpump, 2011). At the same time, the incidence of thyroid nodules and thyroid cancer is increasing, causing varying degrees of burden to both patients and society (Huang et al., 2023; Uppal et al., 2023), and thyroid cancer is the most common endocrine malignancy, accounting for 1% of cancers in the entire human population (Philippe & Dibner, 2015).

Sleep is a physiological restorative process that is critical to human health, maintaining and influencing the performance of all physiological functional roles of the body (Steiger, 2003; Zielinski et al., 2016). According to surveys, millions of Americans suffer from sleep problems each year, with only half of the total number of people maintaining a healthy sleep duration (7–9 h) (Covassin & Singh, 2016), and the increasing severity of insomnia exists in other countries as well (Bin et al., 2012). Results of a study examining the relationship between sleep duration and thyroid function showed a negative correlation between sleep duration and serum free triiodothyronine (FT3) levels (Wang et al., 2023), and several studies have observed that thyroid stimulating hormone (TSH) secretion is affected by diurnal variation (Ehrenkranz et al., 2015; Roelfsema & Veldhuis, 2013). A meta‐analysis examining insomnia symptoms as a predictor of cancer showed that patients with insomnia symptoms had a 24% increased risk of cancer compared to patients without insomnia, which was only significant for thyroid cancer, and that women with insomnia symptoms had a higher risk than men (Shi et al., 2020).

This study intends to assess the bidirectional causal association between insomnia and hyperthyroidism, hypothyroidism, thyroiditis, thyroid nodules, and thyroid cancer because the relationship between thyroid illness and insomnia is currently unclear.

## 2 MATERIALS AND METHODS

### 2.1 Study design

**FIGURE 1:**
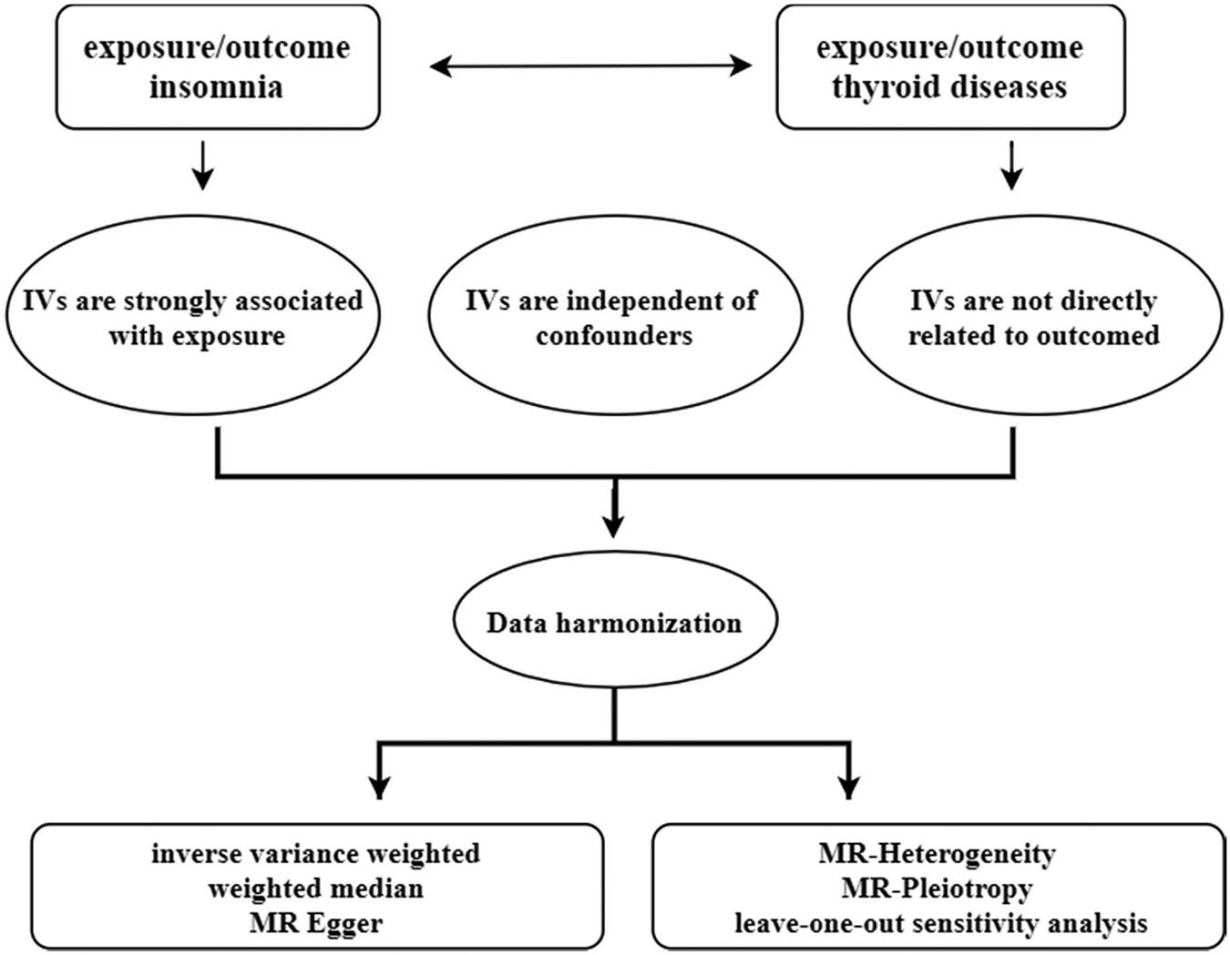
Workflow of the Mendelian randomization (MR) study. IVs, instrumental variables.

The present study used Mendelian randomization (MR) analysis, which overcomes the problem of bias in other research methods by searching for exposures using genetic variation, to determine the causal relationship between exposure and outcome (Smith & Ebrahim, 2003, 2004). In order to evaluate the bidirectional causal link between insomnia and five thyroid illnesses, our study employed a two‐sample MR analysis (Figure 1). No further ethical approval was needed for this work because the data were primarily reanalyzed from previously published data that were retrieved from public sources. Additionally, our findings were presented in compliance with the MR‐STROBE criteria (Skrivankova et al., 2021).

### 2.2 Data sources

Insomnia data were obtained from the UK Biobank Sleep Traits GWAS: Self‐report (insomnia associations); these data have been made public (http://www.kp4cd.org/softet_downloads/sleep). Genetic associations were obtained by genome‐wide association analysis for a total of 1331,010 individuals from the UK Biobank (*N* = 386,533) and 23 and Me (*N* = 944,477) (Jansen et al., 2019). Data on thyroid disorders were all obtained from the FinnGen Consortium version R9 (Kurki et al., 2023). Data for hyperthyroidism were from 367,578 individuals (5590 cases and 361,988 controls); data for hypothyroidism were from 314,995 individuals (40,926 cases and 274,069 controls); data for thyroiditis were from 369,160 individuals (1753 cases and 367,407 controls); and data for thyroid nodules (nontoxic goiter/thyroid nodules) from 377,277 individuals (9485 cases and 367,792 controls); and thyroid cancer from 288,920 individuals (1783 cases and 287,137 controls). In our MR analysis data sources, the participants were predominantly of European origin (Table 1).

**TABLE 1:** Details of the GWAS summary‐level data.

Traits	*N* case	Sample size	Consortium	Data accession address
Insomnia	288,557	1331,010	23 and Me and UKB	http://www.kp4cd.org/softet_downloads/sleep
Hyperthyroidism	5590	367,578	Finngen	https://r9.finngen.fi/
Hypothyroidism	40,926	314,995	Finngen	https://r9.finngen.fi/
Thyroiditis	1753	369,160	Finngen	https://r9.finngen.fi/
Thyroid nodule	9485	377,277	Finngen	https://r9.finngen.fi/
Thyroid cancer	1783	288,920	Finngen	https://r9.finngen.fi/

### 2.3 Selection of instrumental variables

The following conditions had to be met for genetic variants to be considered instrumental variables (IVs) in this study (Gagliano Taliun & Evans, 2021): (1) the IVs had to have a strong correlation with sleep; (2) the IVs had to be unrelated to both insomnia and confounders linked to thyroid disorders; and (3) the IVs had to be associated with insomnia outcomes only, having no direct correlation with thyroid disorders and only impacting thyroid disorders indirectly through insomnia. In order to incorporate more SNPs related to insomnia, we first identified SNPs that were highly associated with insomnia from published data and adopted a more lenient threshold (*p* < 1E − 5) (Lv et al., 2021). We eliminated SNPs that were in linkage disequilibrium (LD) (*r* ^2^ < .001, kb = 10,000) (Machiela & Chanock, 2015) and further eliminated through the PhenoScanncer database (http://www.phenoscanner.medschl.cam.ac.uk/) confounding factors in order to guarantee the independence of the instruments used for exposure. Next, we took five IVs from the GWAS for thyroid disease and applied them to the previously mentioned insomnia. Ultimately, we were able to eliminate palindromic SNPs by finding a match between the SNP's influence on exposure and its effect on outcome. This was achieved by balancing the exposure and outcome data. To remove bias brought about by weak IVs in the data, we additionally computed the *F*‐statistic. If the statistical *F* value was greater than 10, we regarded SNPs as strong IVs with a minor, weak instrumental bias (Burgess & Thompson 2017). All of the pertinent IVs in the current MR investigation had *F*‐statistics larger than 10, which suggests that they are less vulnerable to weak IV bias.

### 2.4 Data analysis

In this study, the results of three MR methods—inverse variance weighted (IVW), weighted median, and MR‐Egger—were used as the primary metrics. Among them, IVW is the main research method, which is an extension of the Wald ratio estimator based on the principle of meta‐analysis (Pagoni et al., 2019). The other two methods were used as complementary methods, where the criterion for the weighted median method is that at least 50% of the SNPs must fulfill the prerequisite that they are valid IVs (Bowden et al., 2016). The weighted median method is less powerful for testing causal effects but also less biased (Hartwig et al., 2017). In contrast to IVW, the MR‐Egger method takes into account the presence of an intercept term, and when horizontal pleiotropy is present, the MR‐Egger method also provides an assessment of bias (Burgess et al., 2017).

For the sensitivity analysis in this study, Cochran's *Q* test in mr_heterogeneity was first used for detecting heterogeneity, and Cochran's *Q* test is mainly used to explore heterogeneity due to multiplicity or other reasons (Greco et al., 2015). Horizontal multiplicity was then tested using mr_pleiotropy. Finally, sensitivity analyses were performed using the leave‐one‐out method, and after excluding 1 SNP at a time, the remaining SNPs were analyzed again as exposed genes for IVW effects in order to determine the influence of individual SNPs on the analysis results and assess the stability of the study results. Analyses were performed by the software package TwoSampleMR in R (version 4.3.1).

## 3 RESULTS

### 3.1 Results of MR analysis

The MR results in this section are based on IVs screened at the genome‐wide significance threshold (*p* < 1E − 5), and we assessed the causal effect of insomnia on thyroid disease based on 13 IVs after performing a LD screen (*r* ^2^ < .001, kb = 10,000) and excluding outcome‐related SNPs. In addition, the *F*‐statistics for all IVs ranged from 29.75 to 50.68.

**FIGURE 2:**
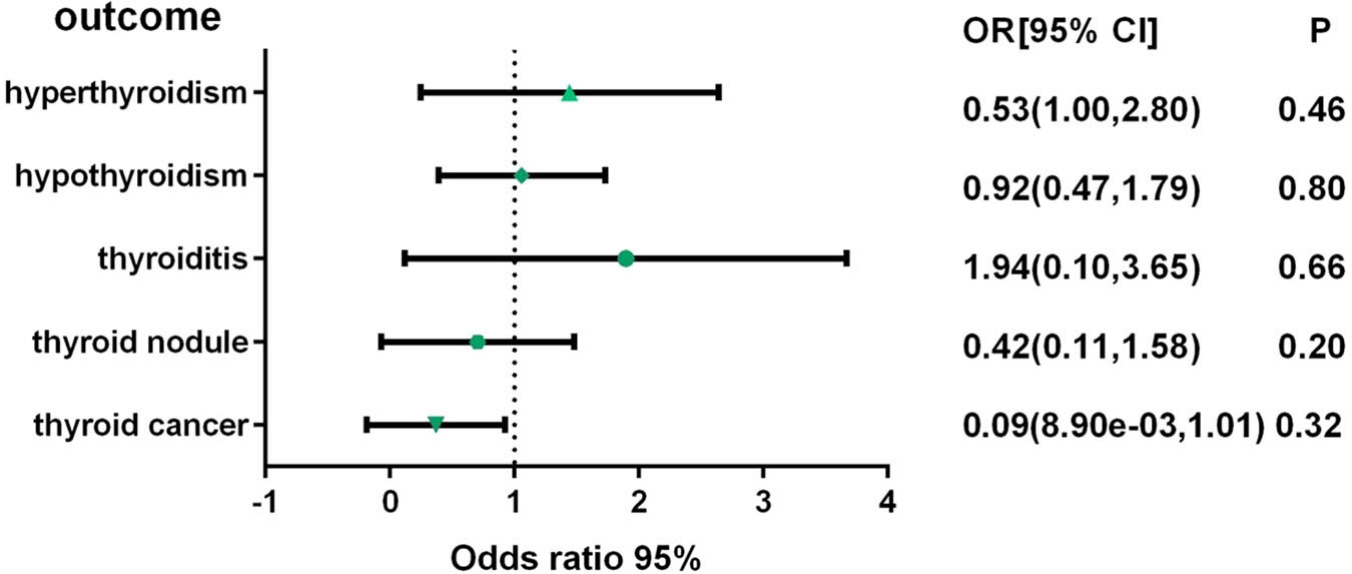
Forest plot of Mendelian randomization estimates for effects of insomnia on risk of thyroid diseases. Estimates were obtained using the random‐effects inverse‐variance weighted method. CI, confidence interval; OR, odds ratio.

The MR results showed no causal association (*p* > .05) between insomnia and the risk of hyperthyroidism, hypothyroidism, thyroiditis, thyroid nodules, and thyroid cancer (Figure 2, Table S1). In addition, similar causal estimates were derived from the three MR methods: IVW, weighted median method, and MR‐Egger method.

The results of the sensitivity analysis indicated that there was no heterogeneity among the IVs (*p* > .05) (Table 2). In addition, the results of the mr_pleiotropy horizontal polytropy test indicated that the MR analysis was not affected by any potential effect of horizontal polytropy (*p* > .05) (Table 2). Finally, the leave‐one‐out sensitivity analysis confirmed the robustness of the MR results, as there were no major SNPs that could significantly affect the results after exclusion (Figures S1 and S2).

**TABLE 2:** Heterogeneity and pleiotropy test results of insomnia on thyroid diseases.

Outcomes	Method	Heterogeneity		Pleiotropy	
		*Q*	*p*	Intercept	*p*
Hyperthyroidism	MR‐Egger	6.59	.47	−0.01	.88
	IVW	6.62	.58		
Hypothyroidism	MR‐Egger	9.28	.32	0.01	.60
	IVW	9.62	.38		
Thyroiditis	MR‐Egger	2.50	.93	0.11	.23
	IVW	4.24	.84		
Thyroid nodule	MR‐Egger	8.39	.40	−0.03	.23
	IVW	10.14	.34		
Thyroid cancer	MR‐Egger	1.14	.56	−0.01	.97
	IVW	1.15	.77		

Abbreviations: IVW, inverse variance weighted; MR, Mendelian randomization.

### 3.2 Results of inverse MR analysis

We performed a reverse MR analysis to assess whether the five thyroid disorders affect sleep. First, the causal relationship with insomnia was assessed for each of the five thyroid disorders after excluding SNPs that were absent from the SNP results associated with the five thyroid disorders, SNPs associated with the results, and the retrospective SNPs. In addition, the range of *F*‐statistics for all IVs was 19.51–33.62.

**FIGURE 3:**
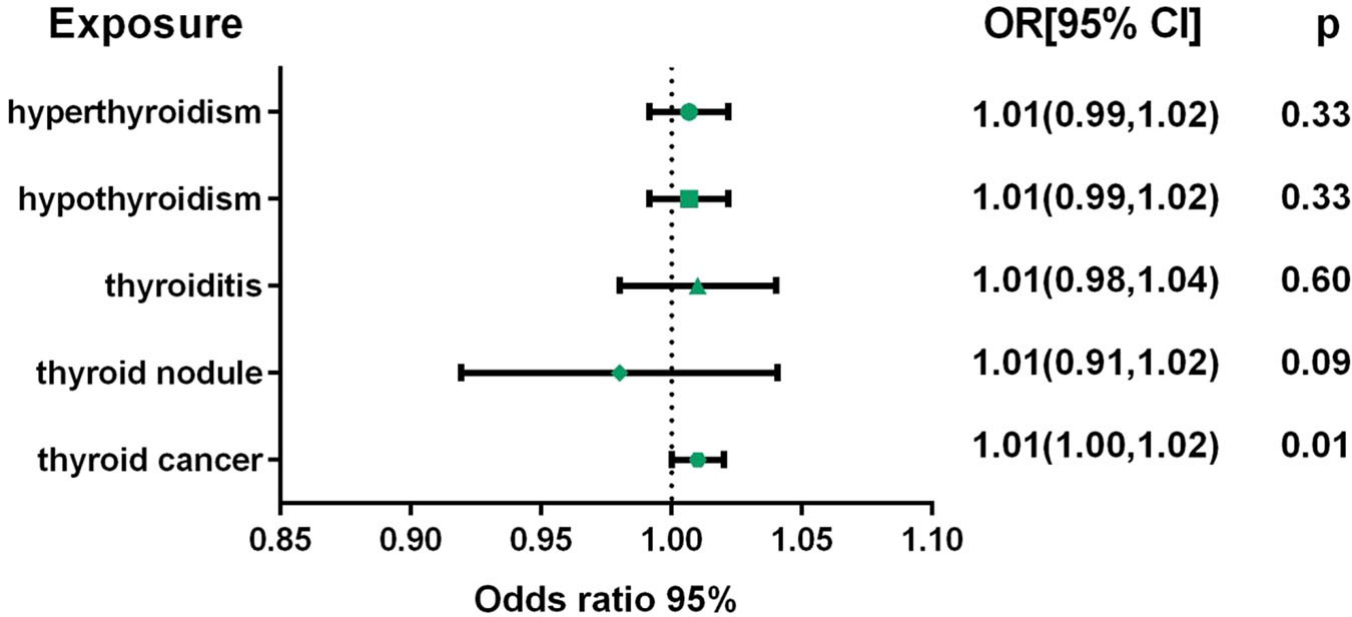
Forest plot of the Mendelian randomization estimates for effects of thyroid diseases on risk of insomnia. Estimates were obtained using the random‐effects inverse‐variance weighted method. CI, confidence interval; OR, odds ratio.

The MR results showed that thyroid cancer significantly increased the risk of insomnia (OR = 1.01, 95%CI: 1.00–1.02, *p* = .01), and there was no causal relationship among the four thyroid disorders, namely, hyperthyroidism, hypothyroidism, thyroiditis, and thyroid nodules, and the risk of insomnia (*p* > .05) (Figure 3, Table S2). The results of sensitivity analysis showed that reverse MR analysis was not affected by heterogeneity (*p* > .05) (Table 3). In addition, the results of the horizontal polytropy test showed that the reverse MR analysis was not affected by horizontal polytropy (*p* > .05) (Table 3). Finally, the leave‐one‐out sensitivity analysis confirmed the robustness of the reverse MR results (Figures S3 and S4).

**TABLE 3:** Heterogeneity and pleiotropy test results of thyroid diseases on insomnia.

Exposure	Method	Heterogeneity		Pleiotropy	
		*Q*	*p*	Intercept	*p*
Hyperthyroidism	MR‐Egger	4.21	.53	0.01	1.34
	IVW	5.49	.46		
Hypothyroidism	MR‐Egger	4.74	.69	−0.01	.39
	IVW	5.59	.69		
Thyroiditis	MR‐Egger	2.26	.52	0.01	.68
	IVW	2.47	.65		
Thyroid nodule	MR‐Egger	4.11	.39	−0.01	.52
	IVW	4.62	.46		
Thyroid cancer	MR‐Egger	2.35	.31	−0.01	.95
	IVW	2.36	.50		

Abbreviations: IVW, inverse variance weighted; MR, Mendelian randomization.

## 4 DISCUSSION

We used a two‐sample MR approach to thoroughly examine the question of whether insomnia influences the incidence of thyroid disorders, including hyperthyroidism, hypothyroidism, thyroiditis, thyroid nodules, and thyroid cancer. No clear evidence was found to support a genetic prediction of the causal role of insomnia in any of the five thyroid disorders. The reverse MR analysis only found a significant causal relationship between thyroid cancer and insomnia and found no causal relationship for the other thyroid disorders.

Thyroid disorders and insomnia are common clinical conditions, and we found clinical overlap between hyperthyroidism and hypothyroidism and sleep conditions by reviewing studies assessing the relationship between thyroid disorders and insomnia, findings that emphasize the importance of monitoring thyroid function in patients with insomnia (Green et al., 2021). The secretion and conduction of thyroid hormones play a key role in human growth and development and have varying degrees of influence on a wide range of physiological processes in the body (Yen, 2001). In addition, the production of most hormones has a circadian rhythm with 24‐h intervals, and sleep affects the regulation of this circadian rhythm to varying degrees (Yuen et al., 2019). Interestingly, sleep has a significant impact on TSH levels, and FT3 follows a circadian pattern that is compatible with TSH (Ehrenkranz et al., 2015; Russell et al., 2008). Research has indicated that hormones and sleep play a critical role in human physiological processes and that sleep and the endocrine system interact (Dijk & Landolt, 2019; Steiger et al., 1998). It is true that thyroid hormone production is significantly influenced by sleep, and thyroid hormone levels in turn impact the quality of sleep. TSH, triiodothyronine (T3), and thyroxine (T4) levels were found to be directly correlated with the intensity of sleeplessness symptoms in one study (Xia et al., 2013). The association between untreated subclinical hypothyroidism and insomnia has been reported numerous times. Song et al. (2019) discovered that people with low TSH levels often have poorer sleep quality and longer sleep latency than people with normal thyroid function. A systematic evaluation study found that a decrease in sleep quality and duration was positively associated with subclinical thyroid function decline, but forward‐looking studies are needed to further clarify the relationship between the two (Teliti et al., 2024). Additionally, a number of retrospective cohort studies and systematic review studies on insomnia and cancer risk show that having symptoms of insomnia could raise the risk of thyroid cancer (Benz et al., 2023; Yoon et al., 2023).

Nevertheless, there is no proof in our study that insomnia contributes to the elevated risk of any of the five thyroid conditions. Our findings were interpreted in light of additional variables. First, self‐reporting without strict rating criteria has been the basis for participant sleep duration in studies on the relationship between thyroid function and sleep; hence, there may be errors between the numbers they provided and their actual sleep duration, and these results can vary due to recall bias and different seasons. Anxiety and depression are two mental diseases that have been linked to insomnia in previous research (Cox & Olatunji, 2020). In other words, insomnia can contribute to the development of anxiety and sadness. In addition, it has been shown that anxiety and depressive mood may cause thyroid dysfunction (Zhu et al., 2023), which is also a risk factor for depression (O'Connor et al., 2023). In addition, we know that the prevalence of thyroid disease is much higher in women than in men (Shi et al., 2020), and one of the reasons for this is that adverse emotions such as anxiety and depression are more present in women. Thus, the mechanism of the link between thyroid disease and insomnia may involve some interaction between both components. This suggests that although insomnia may not be directly linked to a higher risk of thyroid disease, it may have an indirect effect due to psychological variables like depression and anxiety. Although the majority of research indicates that insomnia is prevalent among people with thyroid disease, it might not be the only symptom.

We also found no evidence of a causal relationship between the four thyroid diseases other than thyroid cancer and insomnia. Poor sleep quality in thyroid patients may be the result of a combination of factors rather than the disease itself. Anxiety and depression are prevalent in patients with thyroid disorders, and those with anxiety and depression will be more likely to experience insomnia compared to normal individuals (Difrancesco et al., 2019). Treatment of thyroid cancer can lead to very high levels of physical anxiety and prognosis worries in patients, both physically and mentally, leading to insomnia. Above all, side effects caused by cancer‐related treatments may also lead to anxiety and depression, affecting the quality of sleep in patients (Dahiy et al., 2013). Second, studies have shown that tumor and antitumor treatments promote the secretion of proinflammatory cytokines, which act on the central nervous system and thus negatively affect sleep (Savard et al., 2009). However, studies have shown that patients with thyroid cancer who receive treatment and those who undergo long‐term follow‐up, especially those with differentiated thyroid cancer, do not exhibit greater sleep disturbances compared to patients who undergo benign lesions after thyroidectomy (He et al., 2015). So the relationship between thyroid cancer and insomnia requires further, larger, and more in‐depth research to confirm and explain the physiological mechanisms in detail.

We omitted smoking and alcohol intake as confounders while manually scanning the PhenoScanner database for SNPs to employ in the study because they have been demonstrated to be common predictors of sleep and thyroid issues (Gibson et al., 2019; Hu et al., 2020; Jiang et al., 2023). Even when the confounding SNPs were eliminated, the study's results remained unchanged. This could be because there were just a few confounding SNPs that we uncovered that altered pleiotropy and did not yet affect the outcomes.

The MR results of this study suggest that thyroid cancer increases the risk of insomnia. The prevalence of sleep disorders in cancer patients is at least twice as high as in the general population (Berger, 2009). A study on quality of life in thyroid cancer patients showed that fatigue and insomnia were the two most common symptoms (Alsaud et al., 2023), and the results of several previous observational studies are consistent with the results in this study. Sleep quality is an important factor in the quality of life of cancer patients (Mercadante et al., 2004). Sleep deprivation can lead to physical fatigue during the day, mood disorders, impairment of memory, and concentration, which can prevent the performance of daily activities or even increase pain and compromise the patient's immune system (Berger et al., 2007; Roscoe et al., 2007; Savard & Morin, 2001). Some studies have shown the presence of sleep disorders in patients with thyroid cancer, which may be related to 131I treatment and psychological fear of disease progression (He et al., 2015). In conclusion, sleep in patients with thyroid cancer needs extra attention in clinical practice.

The benefits of the current study are as follows: First, this study greatly expanded the body of research on the topic of insomnia and thyroid problems because, to the best of our knowledge, it is the first to use MR analysis to examine the causal association between five thyroid abnormalities and insomnia in a bidirectional manner. Second, compared to other observational studies on insomnia and thyroid diseases, the study's MR design was less prone to confounding variables. Third, the IV employed in this work was sufficiently statistically valid to estimate causality because it came from a large GWAS database. Furthermore, the study's conclusions are more credible because heterogeneity and horizontal pleiotropy were identified and adjusted for utilizing the MR‐heterogeneity and MR‐pleiotropy methodologies.

However, our research has certain shortcomings. First, because the individuals in the GWAS dataset that we utilized were European by nationality. As a result, it is uncertain what will happen if we expand our investigation to other areas. Second, even though the outcomes of our sensitivity analysis revealed no pleiotropy, the MR analyses did not entirely exclude multiple effects. In conclusion, neither subgroup analysis nor classification of insomnia based on sleep duration or features was done in our study. In order to further elucidate the connection between thyroid illness and insomnia, additional genetic data and large sample studies are required in the future.

## AUTHOR CONTRIBUTIONS

**Zhonghui Li**: Writing—review and editing; writing—original draft; conceptualization. **Zonghang Jia**: Software; methodology; writing—review and editing. **Peng Zhou**: Visualization; software; writing—review and editing. **Qingqing He**: Writing—review and editing; supervision; funding acquisition.

## CONFLICT OF INTEREST STATEMENT

The authors declare no conflicts of interest.

### PEER REVIEW

The peer review history for this article is available at https://publons.com/publon/10.1002/brb3.70046.

## Supporting information



Supporting Information

## Data Availability

The original contributions presented in the study are included in the article/Supporting Information section. Further inquiries can be directed to the corresponding author.
