# Supplementary material for: Causal relationship between insomnia and thyroid disease: A bidirectional Mendelian randomization study
Source: Brain Behav. 2024 Sep 18;14(9):e70046. doi: 10.1002/brb3.70046 (PMC11410884; doi:10.1002/brb3.70046)
Supplement: Supplementary file 1 — Supporting Information [file BRB3-14-e70046-s001.docx]

Supplementary data

Table 1 Results of the causal effect of insomnia on thyroid diseases.

| Outcomes | N SNPs | Method | OR（95%CI） | p |
| --- | --- | --- | --- | --- |
| hyperthyroidism | 10 | IVW | 0.53(1.00,2.80) | 0.46 |
|  |  | Weighted median | 0.79(7.84,7.91) | 0.84 |
|  |  | MR-Egger | 1.32(1.97e-05  ,88919.24) | 0.96 |
| hypothyroidism | 11 | IVW | 0.92(0.47,1.79) | 0.80 |
|  |  | Weighted median | 0.94(0.41,2.78) | 0.88 |
|  |  | MR-Egger | 0.41(0.02,8.15) | 0.58 |
| thyroiditis | 10 | IVW | 1.94(0.10,3.65) | 0.66 |
|  |  | Weighted median | 1.02(2.60e-02,40.32) | 0.99 |
|  |  | MR-Egger | 4.28e-06(1.32e-14,1392.67) | 0.26 |
| thyroid nodule | 11 | IVW | 0.42(0.11,1.58) | 0.20 |
|  |  | Weighted median | 0.31(0.05,1.73) | 0.18 |
|  |  | MR-Egger | 13.43(0.06,2969.75) | 0.37 |
| thyroid cancer | 4 | IVW | 0.09(8.90e-03,1.01) | 0.32 |
|  |  | Weighted median | 0.14(5.57e-04,3.31e+01) | 0.48 |
|  |  | MR-Egger | 0.42(3.10e-33,5.75e+31) | 0.98 |

Table 2 Results of the causal effect of thyroid diseases on insomnia.

| Exposure | N SNPs | Method | OR（95%CI） | p |
| --- | --- | --- | --- | --- |
| hyperthyroidism | 5 | IVW | 1.01(0.99,1.02) | 0.33 |
|  |  | Weighted median | 1.01(0.99,1.03) | 0.36 |
|  |  | MR-Egger | 0.99(0.95,1.03) | 0.53 |
| hypothyroidism | 12 | IVW | 1.01(0.99,1.02) | 0.33 |
|  |  | Weighted median | 1.01(0.99,1.03) | 0.35 |
|  |  | MR-Egger | 1.03(0.98,1.09) | 0.29 |
| thyroiditis | 6 | IVW | 1.01(0.98,1.04) | 0.60 |
|  |  | Weighted median | 0.99(0.96,1.03) | 0.75 |
|  |  | MR-Egger | 0.99(0.89,1.09) | 0.79 |
| thyroid nodule | 7 | IVW | 1.01(0.91,1.02) | 0.09 |
|  |  | Weighted median | 1.01(0.10,1.02) | 0.16 |
|  |  | MR-Egger | 1.02(0.99,1.04) | 0.26 |
| thyroid cancer | 4 | IVW | 1.01(1.00,1.02) | 0.01 |
|  |  | Weighted median | 1.01(0.10,1.02) | 0.10 |
|  |  | MR-Egger | 1.01(0.10,1.03) | 0.26 |


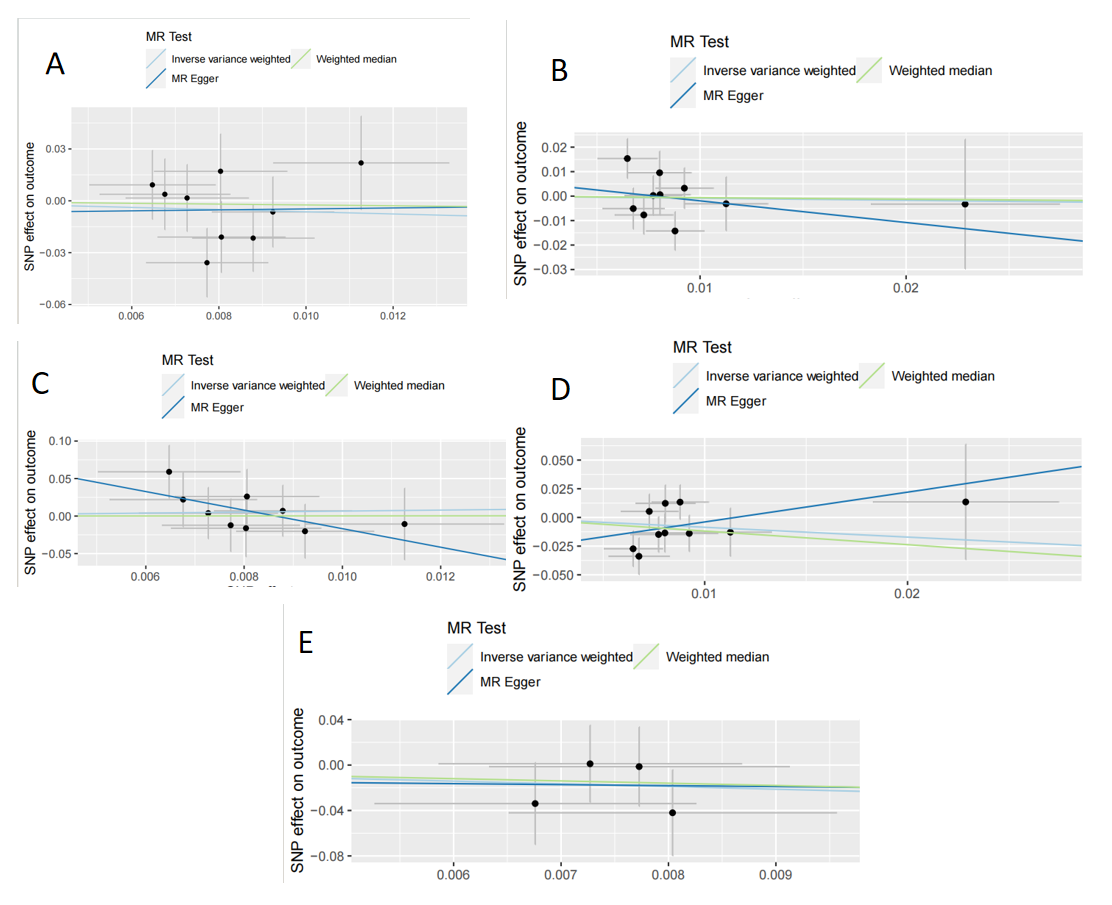


Figure1 Scatter plot of the association between insomnia and thyroid diseases. (A)hyperthyroidism ; (B)hypothyroidism; (C)thyroiditis; (D)thyroid nodule;(E)thyroid cancer. Each black dot represents a SNP, plotted from the SNP estimate for insomnia and the SNP estimate for thyroid diseases risk, with a standard error bar. The slope of the line corresponds to a causal estimate using each of the different methods. SNP, single nucleotide polymorphism.


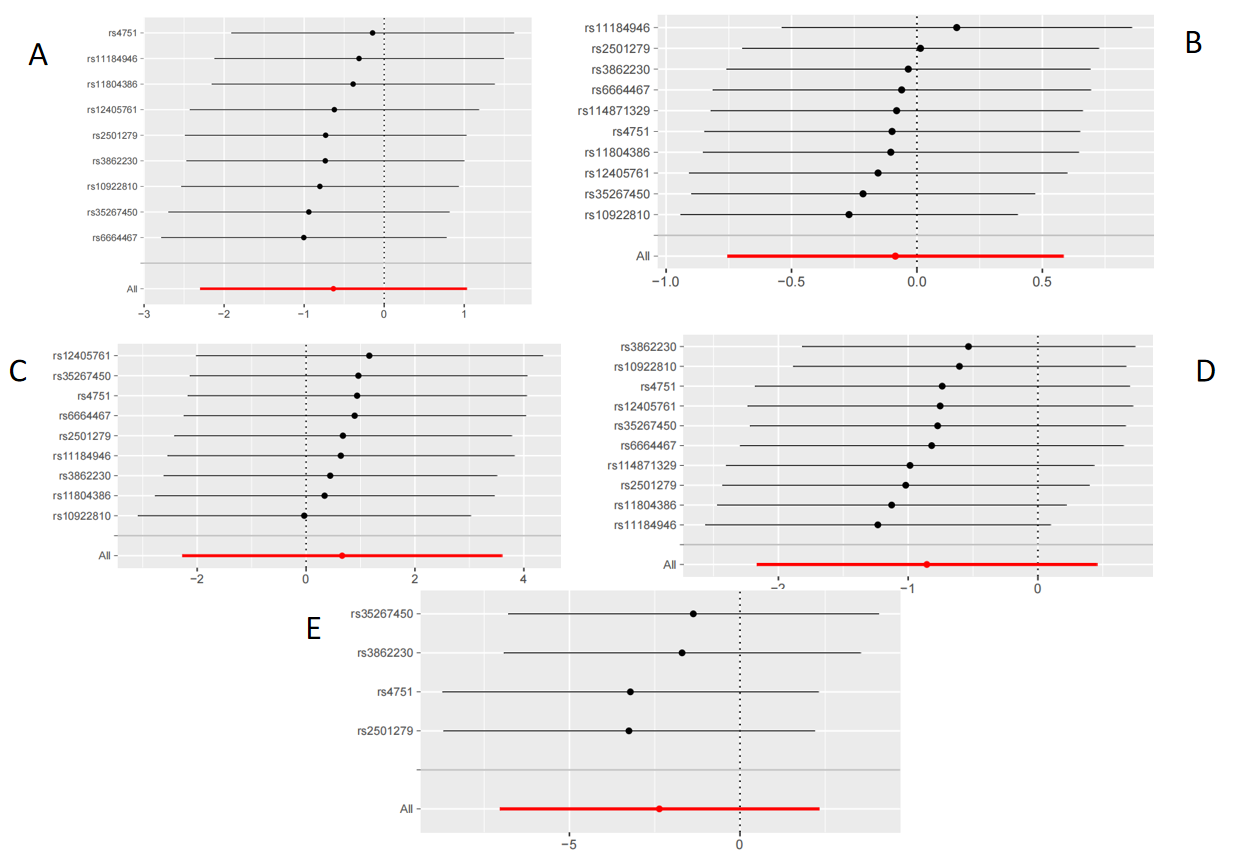


Figure2 Forest plot of the association between insomnia and thyroid disease.(A)hyperthyroidism; (B)hypothyroidism;(C)thyroiditis;(D)thyroid nodule;(E)thyroid cancer.Dots and bars represent causal estimates of the risk of thyroid diseases with insomnia.


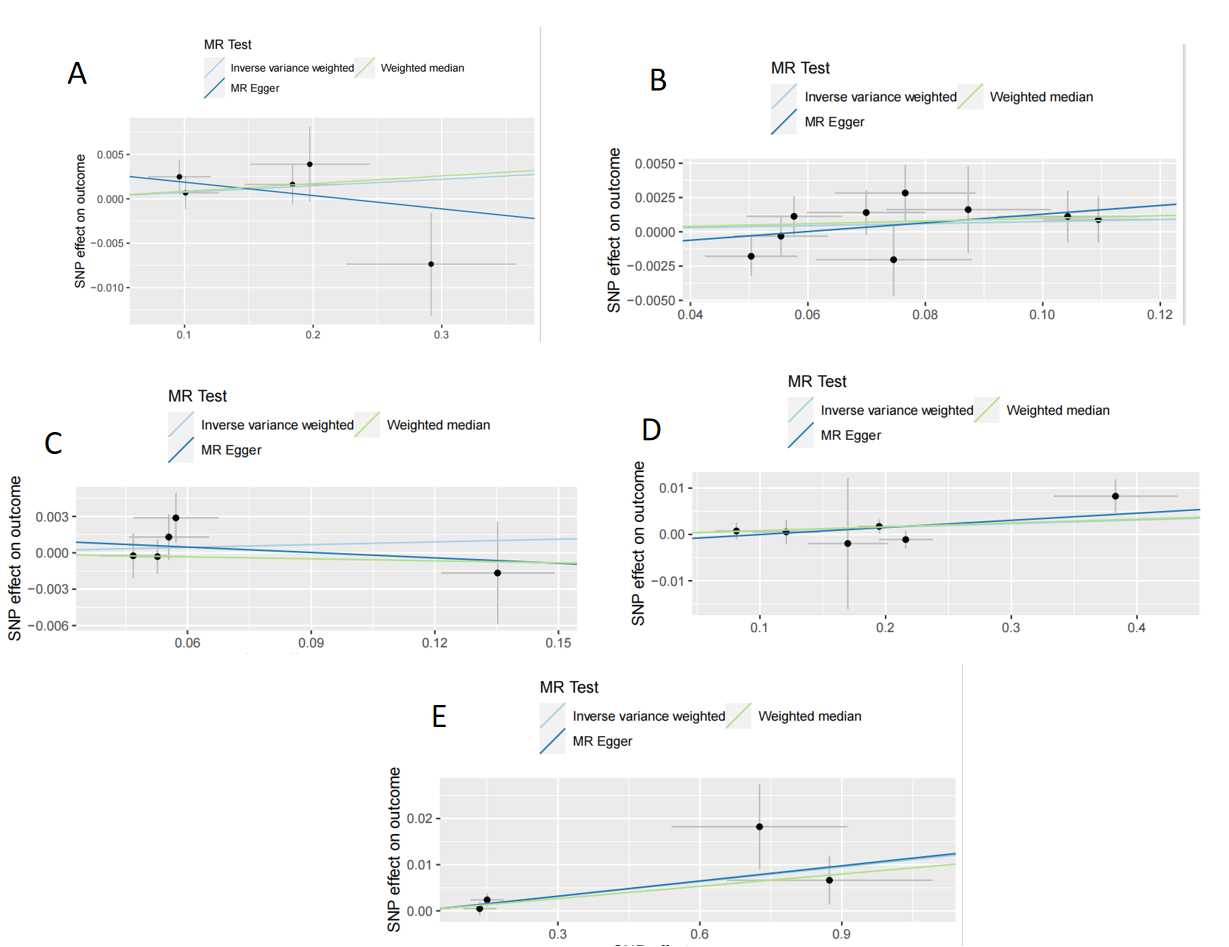


Figure3 Scatter plot of the association between thyroid diseases and insomnia. (A)hyperthyroidism ; (B)hypothyroidism; (C)thyroiditis; (D)thyroid nodule;(E)thyroid cancer. Each black dot represents a SNP, plotted from the SNP estimate for thyroid diseases and the SNP estimate for insomnia risk, with a standard error bar. The slope of the line corresponds to a causal estimate using each of the different methods. SNP, single nucleotide polymorphism.


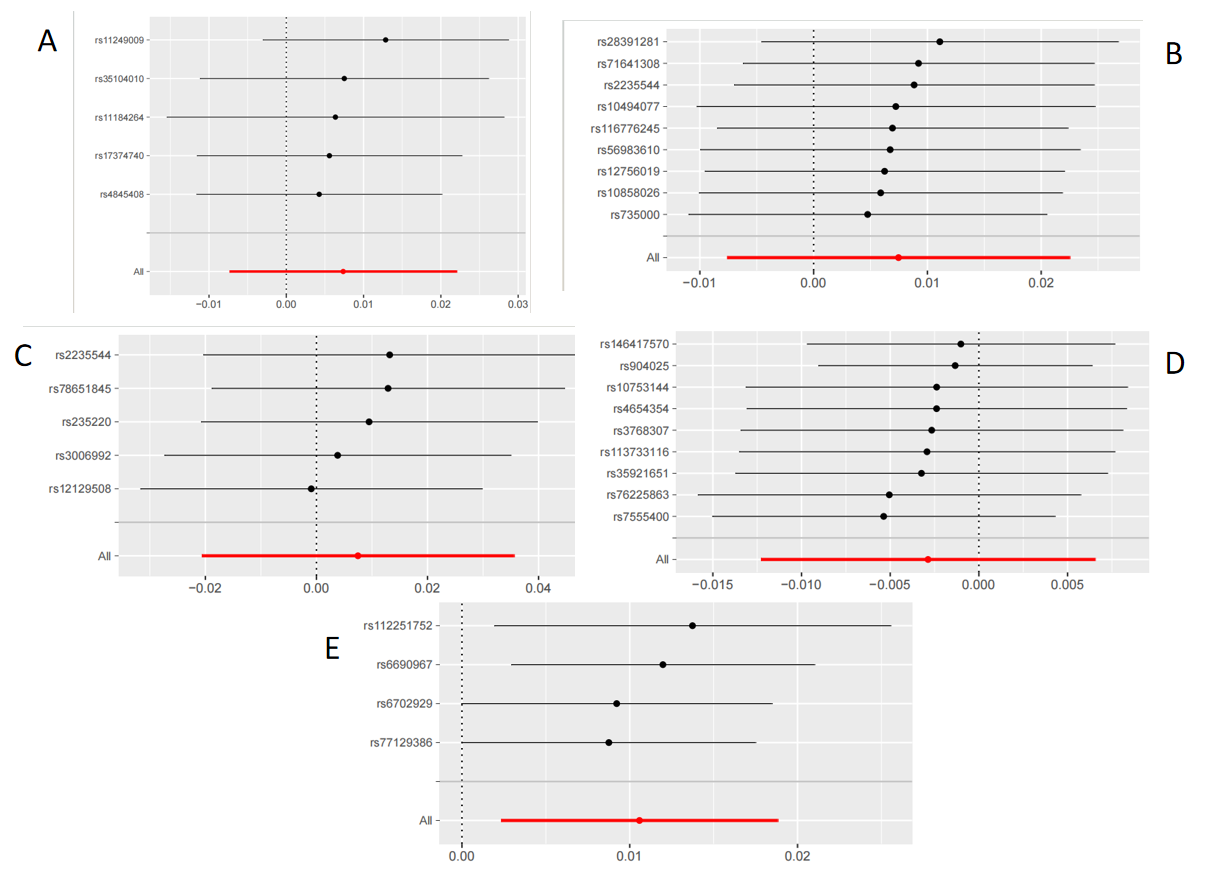


Figure4 Forest plot of the association between thyroid diseases and insomnia . (A)hyperthyroidism; (B)hypothyroidism; (C)thyroiditis;(D)thyroid nodule;(E)thyroid cancer.Dots and bars represent causal estimates of the risk of insomnia with thyroid diseases.
